# Meetings of Societies

**Published:** 1902-12

**Authors:** 


### MEETINGS OF SOCIETIES.

### Bristol Medico-Chirurgical Society.

*Annual Meeting, October 8th, 1902.*

After a vote of thanks to the retiring President (Dr. Barclay J. Baron) had been unanimously passed, Mr. G. Munro Smith took the chair and gave an introductory address (see p. 289), for which a cordial vote of thanks was afterwards given him.

The Honorary Secretary (Mr. J. Paul Bush, C.M.G.) read his Annual Report. The balance in hand was £80 2s. 6d., as compared with £63 7s. 9d. at the end of the previous session. He had to record that the Society had lost three of its members through death; viz., Dr. E. Long Fox, one of the founders of the Society, Dr. Bonville Fox, and Mr. R. H. Chilton. Mr. C. K. Rudge had succeeded Mr. L. M. Griffiths as Librarian. The report was adopted, and thanks given to the Secretary for his services.

The Editorial Secretary read his Report, which was adopted.

The Honorary Librarian of the Medical Library (Mr. C. K. Rudge) gave his Annual Report, and stated that the Society's Library contained 9,898 volumes, including 899 duplicates; 595 volumes had been added to this Library in the course of the year, and 189 periodicals were regularly received. The Bristol Medical Library, of which the former was a part, contained 21,624 volumes, and received 227 periodicals.

The following officers were chosen for the ensuing year: President-elect, Mr. Paul Bush, C.M.G.; Members of Committee, Dr. J. Michell Clarke, Mr. J. Dacre, Dr. B. M. H. Rogers, Mr. J. Taylor, Dr. H. Waldo, and Dr. P. Watson Williams; and members of the Library Committee, Mr. C. K. Rudge, Mr. G. Munro Smith, and Mr. J. Taylor.

---

*November 12th, 1902.*

Mr. G. Munro Smith, President, in the Chair.

Mr. A. W. Prichard showed a "**tumour of the omentum,**" **which he had removed by operation** the preceding day from a female patient. The tumour had been noticed by the patient for two years, and for three weeks had caused a great deal of pain. More than once the patient had lost a good deal of blood from the uterus, and before the operation it had been thought that the tumour was probably a uterine fibroid. The tumour presented as a large mass in the right iliac region; it was well defined, somewhat movable, and peculiarly resonant all over. The hand could be placed between it and the symphysis pubis. Vaginal examination showed the uterus to be situated very high up, and the cervix patulous and directed backwards. Movements given to the tumour were not apparently communicated to the cervix. The tumour had been easily brought outside the abdomen through a free median incision. The intestines were widely adherent to it. It had been removed together with the adherent intestine. The divided ends of the intestine had been united end to end with the aid of O'Hara's forceps. The latter instrument had much facilitated the suturing of the intestine, of which about 49 inches had been removed. The patient up to the present was progressing well.

Dr. A. Ogilvy showed a patient with **congenital subluxation of both crystalline lenses.** He remarked that the affection was supposed to be met with about once in each five thousand cases of eye disease. The patient, a schoolgirl, had been brought to the Eye Dispensary by her mistress, because she could not see well except from the front form in the school. Such displaced lenses were always found to the side of, or to the side of and above, the normal position, never below. In some the lens slipped forward into the anterior chamber, and then there was a risk of glaucoma supervening. Needling the lens was not a satisfactory procedure, as the lens moved about before the needle. It had been suggested to fix the lens with one needle and break it up with another; but the results seemed hardly to warrant interference. The patient had myopia when she looked through the lens, hypermetropia when she looked through the apophyc part. Examined by oblique illumination, the lens looked greyish. It was proposed to leave the case alone.—Mr. CYRIL WALKER thought these cases were actually rarer than statistics appeared to show, because patients with the affection were likely to wander from one institution to another. He mentioned an example of this. With regard to treatment, it should be borne in mind that the lenses were always somewhat opalescent, as seen when the pupil was widely dilated. In one case, in which the lens was widely dislocated, and in which the patient saw by the side of the lens, he had been able to estimate and correct the errors of refraction, but even then sight remained so imperfect that it was obvious that removal of the lens would be of no advantage.—Dr. PROWSE mentioned a case he had seen many years ago in which the lens was dislocated about half-way across the pupil.

Dr. W. H. C. NEWNHAM showed **a specimen of Tumour of the Uterus removed by Intra-peritoneal Hysterectomy.** The specimen presented some peculiarities. The patient was a married woman of thirty years of age, who had had no children. She had been sent to him complaining of an abdominal tumour and profuse uterine hemorrhage. The hemorrhage had been so profuse for eighteen months that the patient was quite blanched. A large tumour, moving with the uterus, was found on examination. The uterus, with the tumour, which was a large fibroid, was removed by retro-peritoneal hysterectomy. For a minute during the operation, when the uterus was cut into, and the tumour bulged out, looking as it did like placental tissue, he had feared that he had incised a pregnant uterus. The patient was now well.—Mr. W. ROGER WILLIAMS remarked that the number of deaths from hemorrhage in cases of uterine fibroids was difficult to decide. It was difficult to collect half a dozen cases. When such tumours had projected into the uterine cavity and broken down, they had been sometimes mistaken for malignant tumours. On the other hand, some such tumours had been reported upon as being innocent, by committees of learned societies, from microscopical examination, and yet recurrence had subsequently shown that the tumours were malignant. Probably the tumour in the present instance had its origin in the fundus of the uterus, where the two lateral halves of the organ became fixed during development.—Mr. Paul Bush asked if the tumour had caused any pressure symptoms, and alluded to a case in which he had to remove such a tumour for intestinal obstruction, due to the pressure it produced. From the symptoms, malignant stricture of the intestine had been surmised.—Dr. Newnham replied that no pressure symptoms had arisen. The tumour had not presented through the cervix.

Mr. J. Taylor read a paper on the "**Clinical Use of High-Frequency Currents**," and showed the apparatus used. The speaker first defined what was meant by the term "high-frequency currents," and demonstrated the different parts of the apparatus used for their production, and the methods of applying the current to obtain therapeutical effects. Cases of phthisis and diabetes had been reported as cured by this agency. The speaker then described some of his own clinical results. He had tried it in three cases of malignant disease. In an epithelioma of the ear and in an epithelioma of the groin, secondary to epithelioma of the vulva, the result had been *nil*. In a case of sarcoma of the orbit, the effects had been encouraging. Pain had been alleviated, the growth had become smaller, the power of hearing, previously lost, had been re-gained, and there had been a slight return of visual power. One patch of lupus in the forearm, which had recurred after treatment by caustics, was cured. A case of paralysis agitans derived benefit. In a case of labyrinthine deafness with tinnitus, the latter symptom had been relieved, but not the deaf-ness. The patient herself was pleased with the result, and came back from time to time to have the treatment re-applied. A man of 80 years of age, with osteo-arthritis, and so much stiff-ness of the shoulder-joints that he could not remove his hat, but without grating in the joints, had been much improved, and he could now dress himself. A man of 50, with obstinate and severe sciatica of three months' duration, had been treated with brilliant success. At first he had to be carried to the room in which the treatment was given. After a few applications he could walk, and after a month could run, and the wasting of muscles previously noted had disappeared. In some simple ulcers the treatment had been successful. In one case, associated with necrosis of bone, the treatment had appeared to the speaker to hasten the separation of a sequestrum and healing of the wound. It had been effectual for a tubercular ulcer in an abdominal scar, the result of a laparotomy for tubercular peritonitis, but on the other hand, on a tubercular sore in an amputation stump, and in a case of tubercular teno-synovitis of the ankle region, the effects had been *nil*. In two cases of fissure of the anus, one associated with hemorrhoids, the fissures had been healed by the treatment, and the hemorr-hoids had been benefited. A case of pruritus ani of twenty years standing was apparently cured.—Dr. Walter Swayne stated that from personal experience he could say that the pain of a stiff neck could be relieved for four or five hours by this treatment, but he could not say that the duration of the trouble had been lessened.—Dr. Kenneth Wills said that all branches of the profession should unite together to settle the question as to the utility of the various forms of electrical treatment now being tested. He was himself convinced that good was being done in various directions, and that the electrical methods of treatment were not destined to fizzle out.—Dr. George Parker agreed with the last speaker. So much quackery had been associated with electrical treatment in the past, that a thorough testing of its value was necessary. It was clear that the improvement in many of Mr. Taylor's cases could not be put down to "suggestion."—Dr. Odery Symes alluded to the alleged inhibitory effect of the currents on the bacillus pyocyaneus. If such effects were produced, then the effect of these currents was quite different to that of X-rays, for the latter had been shown to have no effect whatever on micro-organisms.—Mr. W. Roger Williams asked if Mr. Taylor knew of any case of malignant disease which had been caused by the treatment. Cases had been reported in which X-rays had been supposed to cause the growth of tumours.

Dr. P. Watson Williams described a **Case of Aneurism of the Aorta simulating Mediastinal New Growth,** and showed the specimen which had been obtained from the case. During life he had regarded the case as one rather of mediastinal tumour than of aneurism. The superior vena cava was almost obliterated, being flattened between the aneurismal sac and the chest wall.—Dr. E. Markham Skerritt said that he had not seen the patient. He thought the position of the growth rather pointed to its aneurismal nature. The absence of pulsation in such cases was accidental, and depended upon the size of the mouth of the aneurism. Absence of hypertrophy of the heart was not against aneurism. An aneurism did not give increased work to the heart, and hypertrophy would only be likely to occur if there was coincident valvular disease.—Mr. Roger Williams asked if the pulse had been dicrotic, and if a sphygmographic tracing had been taken.—Dr. Cave alluded to statistics which had been collected, showing that cardiac hypertrophy was not common in aneurism.—Dr. Theodore Fisher described the way mediastinal tumours tended to block veins but not arteries.—Dr. Michell Clarke said that in his experience extreme distension of superficial veins had been present more often in aneurism than in growth. He had only once met with tracheal tugging without an aneurism. He attached great importance to an alteration in the basic heart sounds in the diagnosis of aneurism, and asked if they had been altered in the present case. He had found the greatest difficulty in diagnosing aneurisms affecting the descending aorta, and he mentioned cases in which patients had not had spinal tenderness nor fixation, though vertebræ had been extensively eroded. In one such case death resulted from dysphagia.—Dr. WATSON WILLIAMS replied that in his case there had been no peculiarity in the pulse, no bruit, and no accentuation of the second cardiac sound.

Dr. J. MICHELL CLARKE read a paper on a **Case of Banti's Disease** (to be published in our next number), which was discussed by Drs. SYMES, FISHER, and PARKER.—Dr. SYMES mentioned a case of Banti's Disease which had ended fatally in the Bristol General Hospital in 1901. The blood count was as follows: red cells, 2,800,000; white cells, 6,000 per c.mm. The differential count of the latter showed: finely granular oxyphil leucocytes, 56 per cent.; coarsely granular ditto, 25 per cent.; large lymphocytes, 12 per cent; small lymphocytes, 28 per cent.; and a few granular basophils. The enlarged spleen and liver showed fibrosis only. There was a scar of an old infarct in the lung.

J. LACY FIRTH.
J. PAUL BUSH.

